# Care of Clothing

**Published:** 1891-12

**Authors:** 


### CARE OF CLOTHING.

The proper care of men's as well as women's clothing has a great deal to do not only with its looking well, but with the length of time which it lasts. Clothes of wool, which are rarely brushed and never hung out of doors, soon come to have an appearance of long use, when the same clothes, if carefully brushed every time they are worn and frequently hung out of doors, will always be fresh and will keep their good looks very much longer. Care should be used to select a brush-broom or whisk of fine broom-corn. It will cost more than the coarser ones, but in the end will be a saving, as the coarser ones wear the clothing more rapidly.

Coats and cloaks should always be hung on the little wire frames, costing but five or ten cents, which come for that purpose. The frames should be first covered with soft material to prevent the garments from breaking over their edges. If made of wood this is not necessary ; the wooden ones, however, are a little more expensive. It is better to hang than to fold almost all dresses that are not wash dresses, if one has sufficient room, but if the room is limited and the dresses crowded when hung, then they should be folded, as any thing is better than the " stringy " look which dresses crowded together in a small closet or wardrobe soon acquire. If a dress of a woolen material has any drapery, it will be found to keep its freshness much longer if the skirt is always bottom upwards. With a little practice and care this will be easily done, and the creases prevented which come so quickly even in the best of materials from the folds hanging always the same way, both when in wear and when not.

Never sit in a damp dress if it can be avoided, for nothing so successfully creases it. It should at once be taken off and hung in a good position to dry. Careful attention should always be paid to dress braids and facings. If a braid is replaced as soon as it commences to wear, the facing will in many instances be saved.

A dress braid should always be put on by hand, and in most instances " rolled on." If sewn on by machine, more time is consumed in ripping it off when it requires replacing, than in both sewing on and ripping off a braid sewn on by hand. If one has to be much in the kitchen, woolen dresses should not be worn there. They hold the odors and smoke and soon become grimy and shabby.—*The Housekeeper*.

